# Viruses of Microbes

**DOI:** 10.3390/v9090263

**Published:** 2017-09-20

**Authors:** Laurent Debarbieux, Matthias Fischer, Tessa E. F. Quax

**Affiliations:** 1 Department of Microbiology, Institut Pasteur, F-75015 Paris, France; laurent.debarbieux@pasteur.fr; 2 Department of Biomolecular Mechanisms, Max Planck Institute for Medical Research, 69120 Heidelberg, Germany; mfischer@mpimf-heidelberg.mpg.de; 3 Molecular Biology of Archaea, Institute of Biology II, University of Freiburg, 79104 Freiburg, Germany

Viruses of microbes encompass all viruses that infect archaea, bacteria, and single-celled eukaryotes, especially algae and protozoa. Bacteriophages, the name given by Félix d’Herelle to bacterial viruses, were initially studied for their bactericidal properties to treat infectious diseases, before the discovery of antibiotics. During the second half of the 20th century, these viruses were used as tools to decipher the molecular mechanisms of cellular processes giving birth to molecular biology. Following the golden age of bacteriophage research, microbial viruses, including the more recently discovered viruses of archaea and protists, experienced a renaissance when they were found in many diverse ecosystems. Over the past two decades, the advancement of viral ecology has led to the realization that viruses of microbes are the most abundant biological entities on earth and play a major regulating role in ecosystems. They are recognized as chief contributors to horizontal gene flow and drivers of evolution. In addition, being key predators of cyanobacteria and algae in oceans, they impact global warming and climate change. Thus, viruses of microbes play a pivotal role in evolution, ecology, health, and environmental science.

Only by including the full diversity of viruses that infect all three domains of life can we start to construct a comprehensive image of viral impact in mixed ecosystems. In addition, comparative studies will allow us to address central questions about the definition or classification of viruses.

This special issue was inspired by the Fourth Meeting of the International Society for Viruses of Microbes (ISVM) held in Liverpool in July 2016, and reflects the breadth of microbial viral research that was presented there. It highlights the wide range of recent developments in this field, encompassing both fundamental and applied research.

You will find in this issue several reviews providing an excellent overview of recent scientific advancements on the study of viruses infecting archaea (Demina et al.) [1], algae (van Etten et al.) [2], and bacteria (Manrique et al.) [3]. Despite specific differences between these viruses, similarities in methodology, studied habitats, and molecular mechanisms provide the basis for fruitful exchange between scientists that we hope this special issue will encourage (Torres-Barceló et al.) [4]. The renewed interest to study these viruses, amplified by recent advances in genome sequencing techniques, requires nomenclature guidelines to help naming newly isolated viruses (Adriaenssens et al.) [5], together with the standardization of virus ontology to coordinate exchange between viral databases (Hulo et al.) [6]. Exploiting viral genomic information reveals the diversity of eukaryotic phytoplankton viruses and shows many examples of horizontal gene transfer with bacteria and eukaryotes (Finke et al.) [7]. As metagenomic techniques advance and sequence databases increase, in particular for human samples, viral metagenomics is about to become a powerful tool for ecological and medical diagnoses (Hayes et al.) [8].

Beside genomic information, the isolation of viruses from increasingly diverse environments, is still a prerequisite to perform molecular studies on the multiple facets of the viral infection cycle (Bautista et al.; Mahony et al.; Rashid et al.; Willms et al.) [9,10,11,12]. Such studies culminate with the identification of novel protein functions that often include structural studies (Peeters et al.; Granell et al.; Loh et al.; Decewicz et al.) [13,14,15,16]. Finally, novel applications of phage therapy are being developed, following the historical footsteps of Félix d’Herelle (Sergueev et al.; Majkowska-Skrobek et al.; Jończyk-Matysiak et al.) [17,18,19].

We hope that the articles and reviews included in this special issue will encourage scientists inside and outside this field to intensify research on viruses of microbes, in order to contribute to the immense task of unravelling the molecular mechanisms of viruses in the three domains of life.
